# Emergency management: retinal detachment

**Published:** 2018-11-09

**Authors:** David Yorston

**Affiliations:** 1 Consultant Ophthalmologist: Tennent Institute of Ophthalmology, Gartnavel Hospital, Glasgow, Scotland, UK.

**As life expectancy, cataract surgery and myopia increase, retinal detachment will become more common. Early recognition and referral is essential.**

**Figure 1:**
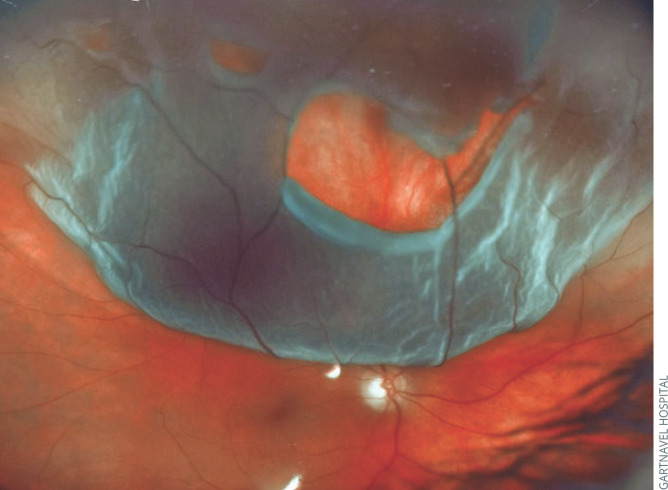
A detached retina, visible as a grey, mobile membrane in the eye

The retina is the light-sensitive part of the eye. The outer layer of the retina is composed of photoreceptor cells, which generate a nerve signal in response to light. Normally, this photoreceptor layer is in close contact with the choroid and retinal pigment epithelium. The photoreceptor cells depend on this contact for their metabolism. If they are separated from the choroid by a retinal detachment, they may be irreversibly damaged. This is why retinal detachment is an ophthalmic emergency.

As the eye ages, the vitreous becomes liquid, and eventually collapses. When this happens, it may pull on the retina, causing a retinal tear. Fluid then passes through the retinal tear and into the potential space between the retina and choroid. This causes a retinal detachment, which may progress rapidly.

The risk factors for retinal detachment are:

- Age (most common in patients aged 50–70)
- Gender (more common in men)
- Cataract surgery, particularly if it was complicated by vitreous loss
- Myopia (more common in longer-sighted eyes).

**“If you recognise it early, and refer immediately, you will save the patient's sight.”**

## Symptoms

The initial symptoms of retinal detachment include a sudden increase in floaters, sometimes with flashes of light in the peripheral vision. As the detachment progresses, there may be a corresponding visual field defect. When the macula detaches, there will be a sudden and severe loss of vision, usually to less than 6/60.

## Clinical signs

- The detachment is visible as a grey, mobile membrane inside the eye (Figure 1)
- Intraocular pressure may be reduced
- The red reflex is usually pale, or grey, rather than the normal orange
- When examining the eye using a slit lamp, you may see pigment cells in the vitreous.

To recognise a retinal detachment, you need to examine the retina. Ensure that you have enough training and practice to develop this skill. Check that the ophthalmoscope works, and that spare bulbs and batteries are available.

## Treatment

Retinal detachment is treated using surgery. The aim of surgery is to close the retinal break. This stops fluid from leaking under the retina and allows it to re-attach. This can be done either by removing the vitreous (using vitrectomy) and filling the eye with a bubble of gas that holds the retina in place, or by stitching a piece of plastic to the sclera (a scleral buckle). This pushes the outer layers of the eye against the break and plugs it. Approximately 85% of retinal detachments can be cured with a single operation. However, the eye will only regain good vision if the operation is carried out soon after the retina detaches.

## Referral

Not all clinics provide retinal surgery, so find out where your nearest retinal detachment surgeon is based. You should be able to explain to the patient how much the treatment will cost, and how to get to the clinic. You should know the contact details of the surgeons so that you can phone to tell them the patient is coming.

If you recognise retinal detachment early, and refer immediately, you will save the patient's sight.

**Be prepared**

- Familiarise yourself with the symptoms and signs of retinal detachment
- Ensure you have an ophthalmoscope that works and that spare batteries and bulbs are available
- Find out where your nearest retinal detachment surgeon is based and keep their contact details that everyone in the clinic can access easily
- Practise examining the retina.

